# Multi-Channel Hyperspectral Fluorescence Detection Excited by Coupled Plasmon-Waveguide Resonance

**DOI:** 10.3390/s131013892

**Published:** 2013-10-14

**Authors:** Chan Du, Le Liu, Lin Zhang, Jun Guo, Jihua Guo, Hui Ma, Yonghong He

**Affiliations:** 1 Shenzhen Key Laboratory for Minimal Invasive Medical Technologies, Graduate School at Shenzhen, Tsinghua University, Shenzhen 518055, China; E-Mails: du-c09@mails.tsinghua.edu.cn (C.D.); guojun@sz.tsinghua.edu.cn (J.G.); guojh@sz.tsinghua.edu.cn (J.G.); mahui@tsinghua.edu.cn (H.M.); 2 Department of Physics, Tsinghua University, Beijing 100084, China; 3 Laboratory of Advanced Power Source, Graduate School at Shenzhen, Tsinghua University, Shenzhen 518055, China; E-Mail: liu.le@sz.tsinghua.edu.cn; 4 Graduate School at Shenzhen, Harbin Institute of Technology, Shenzhen 518055, China; E-Mail: hgd06zl@sina.com

**Keywords:** CPWR excited fluorescence, hyperspectral fluorescence analysis, multi-channel

## Abstract

We propose in this paper a biosensor scheme based on coupled plasmon-waveguide resonance (CPWR) excited fluorescence spectroscopy. A symmetrical structure that offers higher surface electric field strengths, longer surface propagation lengths and depths is developed to support guided waveguide modes for the efficient excitation of fluorescence. The optimal parameters for the sensor films are theoretically and experimentally investigated, leading to a detection limit of 0.1 nM (for a Cy5 solution). Multiplex analysis possible with the fluorescence detection is further advanced by employing the hyperspectral fluorescence technique to record the full spectra for every pixel on the sample plane. We demonstrate experimentally that highly overlapping fluorescence (Cy5 and Dylight680) can be distinguished and ratios of different emission sources can be determined accurately. This biosensor shows great potential for multiplex detections of fluorescence analytes.

## 1. Introduction

Fluorescence technology, since its introduction to biochemistry in the 1950s, has offered an important analytical tool for both biological research and clinical applications in biological molecules [1], DNA microarray technology [2], cell imaging [3], cancer diagnosis [4] and so on [5]. Due to its high sensitivity and selectivity, fluorescence detection has undergone enormous developments in all the aspects of principles [6], instruments [7] and applications [8,9]. Among these achievements, fluorescence-based detection of chemical and biological applications with advanced sensitivity by using surface plasmon-enhanced fluorescence (SPEF) has been a continuous focus of scientific research and led to ever increasing important developments [10,11]. This dark field excitation mode not only increases the intensity of the fluorescence signal greatly, but also avoids the influence of the incident light. It is very useful for the ultra-sensitivity detection of analytes in the range of an enhanced surface evanescent electromagnetic field which is about 200 nm away from the metal surface, but detection of some macromolecular interactions will be limited due to the distance dependency of SPEF. To improve the performance of SPEF, one of the more promising strategies is to generate a guided mode by adding a dielectric layer over a gold or silver film [12,13]. The resonances between guided light modes within the waveguide layer and surface plasmon polarizations occur in a small angular region immediately preceding the critical angle when the dielectric layer is thick enough (λ/2n or greater, where λ is the excitation wavelength and n is the refractive index of the dielectric layer). Coupled plasmon-waveguide resonance (CPWR) offers a narrower full width half-maximum (FWHM) of the reflective curves that leads to increased precision over conventional surface plasmon resonance (SPR) sensors [14]. Meanwhile, theoretical results indicate that CPWR also results in better performance for both detection range and enhanced electromagnetic field which is highly suitable for dark field excitation of fluorescence to maintain an appreciable sensitivity and signal-to-noise ratio [15].

Besides, as the developments in biology and medicine have led to increased throughput [16,17], the capabilities of fluorescence measurements become an important factor that should be considered. Recently developed hyperspectral fluorescence techniques show great potential for multiplexed research projects [18]. The hyperspectral fluorescence detection technique has obtained increasing popularity for detecting fluorophores in DNA sequencing and microarray applications [19,20]. This technology works by recording the entire emission spectra for each pixel of the image via a spectrometer, permitting the capture and identification of different spectral signatures of molecules with partially overlapping emission spectra. Except for its ability to separate the contributions of multiple fluorescent tags and to remove impurity emission, hyperspectral fluorescence detection plays an important role in the determination of the ratios of different emission sources by using multivariate data algorithms [21]. Dependence on custom optical filters and restrictions on the allowable fluorescence labels can also be reduced. Our group has developed this technology by using the method of quasi-confocal and multichannel parallel scanning to analyse multicolor microarrays. The advanced method has exhibited great potential for applications in microarrays [22]. In our previous work [23], we proposed SPR-supporting sensing structures based on symmetrical CPWR and the experimental results showed that the sensing characteristics of this symmetrical CPWR based sensor are greatly improved over conventional SPR sensors (a refractive index resolution of 8.1 × 10^−8^ RIU and 3.5 × 10^−7^ RIU in water and line on the sensor films to cover all the channels of the microfluidic system for fluorescence excitation, and thus multiple channels can be monitored simultaneously at a dark field. Performances of this sensor system are tested and the hyperspectral fluorescence detection allows for quantitative identification of multiple fluorescent dyes and elimination of unnecessary artifacts. It is believed that combinations of hyperspectral fluorescence detection and symmetrical CPWR will exhibit enormous potential in the development of fluorescence technology.

## 2. Materials and Methods

### 2.1. Materials

Dylight680 (absorption/emission: 680/715 nm) and Cy5 (absorption/emission: 649/667 nm) were used as fluorescence tags. Dylight680 AffiniPure Rabbit Anti-Goat IgG (H + L) was purchased from EarthOx, LLC (San Francisco, CA, USA) and 10 μL of the former liquid was diluted in 40 μL of deionized water for use. Cy5 solutions are prepared by diluting 1 mg of Cy5 powder purchased from Zibo Yunhui Bio-Technology Co., Ltd (Shandong, China) in deionized water and then diluting to different concentrations.

### 2.2. Sensor Chips

**Figure 1.**
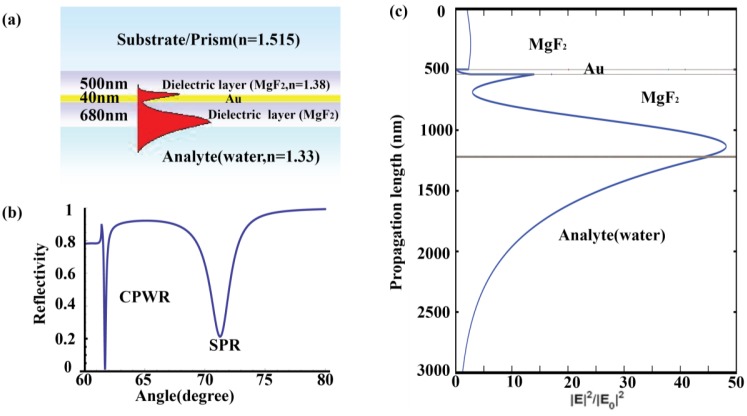
(**a**) 1D implementation of a symmetrical optical waveguide structure based on Kretschmann configuration for CPWR excitation. Theoretically calculated angular reflectivity curves (**b**) and electric field distributions (**c**) for the architecture given in (**a**) at the excitation wavelength of 632.8 nm, n(Au) = 0.3123 + 3.146i. Propagation length in this figure refers to the electric field decay distance away from the sensor surface into the analyte.

Figure 1a shows an example implementation of a symmetrical optical waveguide structure that we utilized in this paper. By using multiple reflectance theory and Frensnel's formulae, the angular distributions of reflectivity (Figure 1b) and evanescent field distributions (Figure 1c) could be calculated for this dielectric-metal-dielectric slab style for the excitation of guided waveguide mode [24]. It can be figured out that the CPWR generated under this symmetrical optical waveguide structure exhibits a sharp resonance dip and an appreciably enhanced electromagnetic field with particularly long propagation length.

According to the theoretical results, three successive layers of MgF_2_-Au-MgF_2_ are deposited directly onto the substrate of a SF_4_ glass for the preparation of sensor films. Polished SF_4_ glass is firstly cleaned with a solution consisting of acetone and ethanol in a 1:1 ratio, dried in a clean air gas stream, then rinsed with deionized water and again dried with nitrogen. The first layer of magnesium fluoride (MgF_2_) is deposited on the glass substrate by vacuum evaporation coating, and a layer of gold film is then deposited by vacuum magnetron sputtering. The chip then is put in a vacuum evaporation facility for the preparation of another MgF_2_ layer. Thicknesses of the MgF_2_ films and gold film are separately measured by ellipsometry (M-2000 UI, J.A. Woollam Co. Inc., Lincoln, NE, USA) and X-ray diffraction (XD-2, Purkinje General, Beijing, China). The sensor chip is attached to a SF_4_ prim with refractive index matching oil for the Kretschmann excitation of coupled plasmon-waveguide resonance.

### 2.3. Experimental Setups

**Figure 2.**
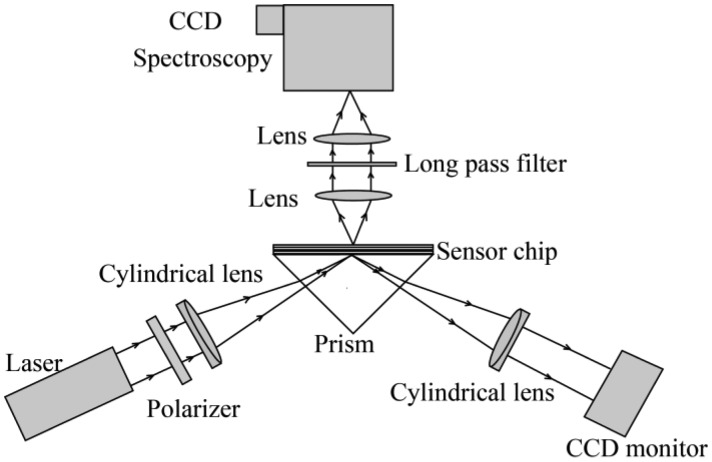
Schematic of the hyperspectral fluorescence detections based on a symmetrical CPWR structure.

The schematic of the system is shown in Figure 2. The excitation light is a He-Ne laser at 632.8 nm with a power of 40 mW. A polarizer is used to produce p-polarized light for the excitation of coupled plasmon-waveguide resonance (CPWR). P-polarized light goes into a cylindrical lens (f = 50 mm), which is used to focus a line-light on the surface of the sensor films.

The laser line covers all the channels of the microfluidic system to implement a simultaneous multi-channel detection. The CPWR excitation makes it possible for the dark-field illumination of fluorescence solutions. The reflectivity curves of the chip are displayed on a CCD monitor. Fluorescence emitted from the line-illuminated area are collected by imaging lenses to image the pattern of the excited line region onto the entrance slit of a spectrometer and this in turn projects a spectrally dispersed image of the line onto a two-dimensional detector array. Each data frame obtained in this manner contains the emission spectra corresponding to all points along the line-illuminated region of the sample. Spectrometer equipped with a reflective grating of 33 lines/mm offers a high spectral resolution of 0.2 nm with a spectral range of more than 200 nm. A long-pass filter (98% reflection at λ = 632.8 nm) is used to further eliminate any unnecessary scattered and reflected laser light.

## 3. Results and Discussion

### 3.1. Sensing Model for Hyperspectra Fluorescence Detection

**Figure 3.**
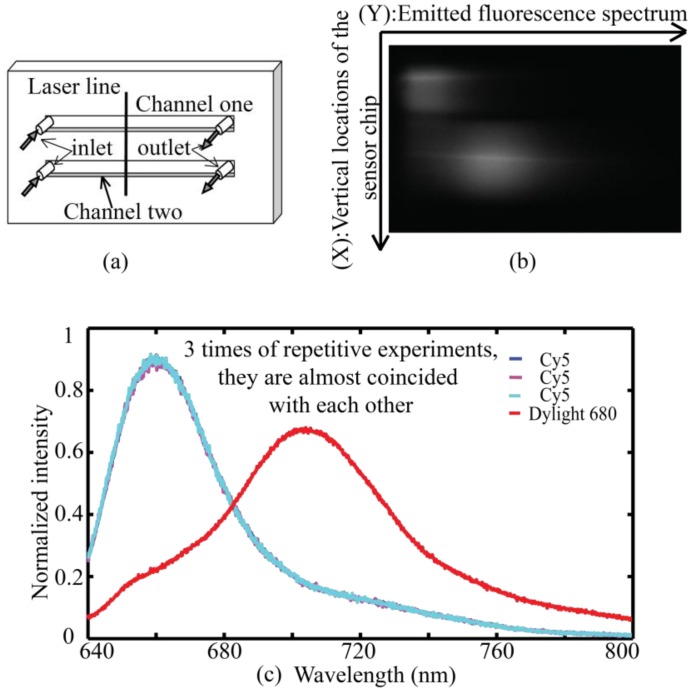
Demonstration of the data processing. (**a**) Structure of the two flow channels and the focused laser line; (**b**) An image captured by CCD, corresponding to the irradiated line region; (**c**) Fluorescence spectra correspond to channel one and channel two separately. Thicknesses for the three successive layers of MgF_2_-Au- MgF_2_ are 500 nm, 40 nm, 600 nm individually.

To demonstrate the data processing, we designed a sensing model for fluorescence detection, as shown in Figure 3a. A two-channel flow cell is mounted against the sensor surface for the detection of fluorescence spectra of Cy5 and Dylight680.

Aqueous samples with a refractive index close to 1.33 are pumped at a flow rate of 0.5 mL/min. The first channel contains solution of Cy5 and the second channel contains solution of Dylight680 labeled Secondary Antibody. Figure 3b shows a spectrally resolved image captured directly by CCD, corresponding to the illuminated line region in Figure 3a. In Figure 3b the horizontal direction represents emitted fluorescence wavelength while the vertical direction corresponds to vertical locations of the sensor chip. We averaged 100 pixels in the middle of the channel in the vertical direction to get fluorescence intensities at each wavelength for every channel. As the spectroscopy allows us to capture the whole spectra of the emitted fluorescence, overlapping fluorescence spectra of Cy5 and Dylight680 can be detected simultaneously by the use of a two-channel flow cell. Spectra corresponding to the two channels are show in Figure 3c. Cy5 solutions with the same concentration are applied to the upper channel and the experiment is repeated three times to demonstrate the repeatability of the experiment.

### 3.2. Characteristics of the Sensor Chips

**Figure 4.**
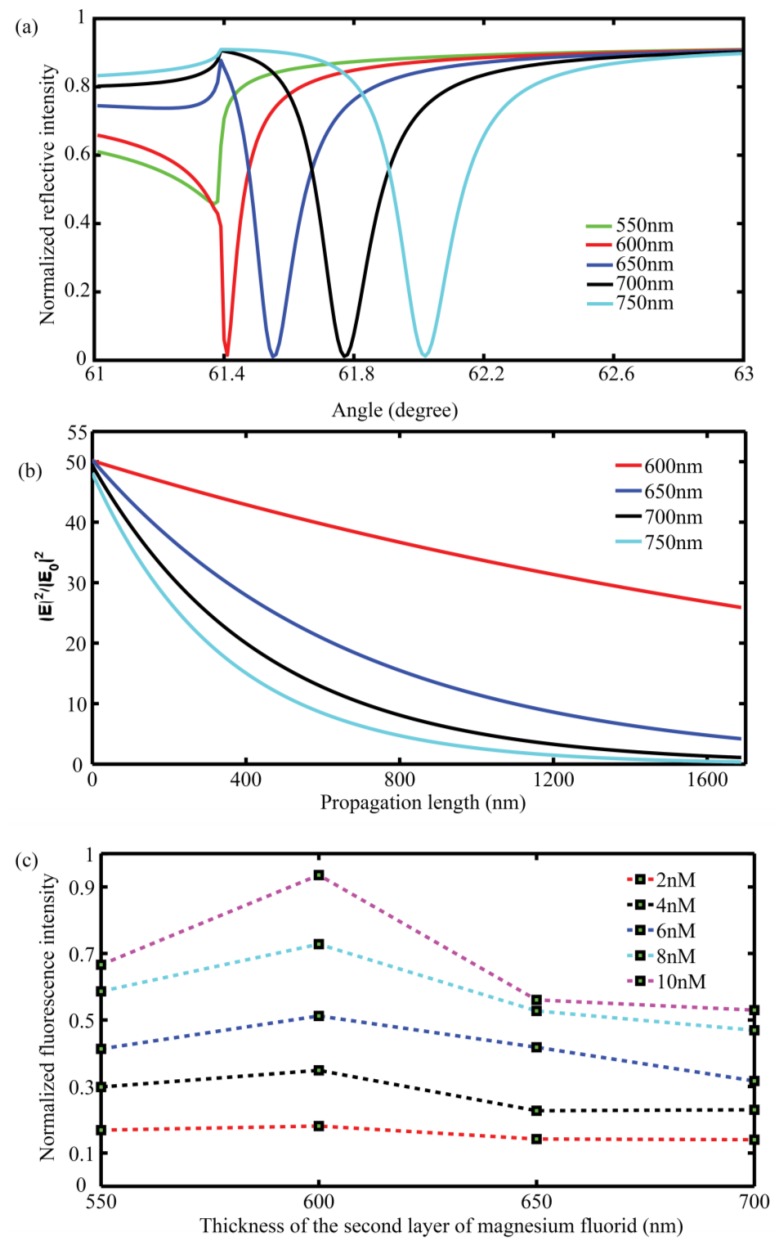
Resonance curves (**a**) and electric field distribution (**b**) beyond the sensor films directly into sensed medium simulated with various thicknesses of second layer of magnesium fluoride 600 nm (red), 650 nm (blue), 700 nm (black), 750 nm (cyanine) generated by symmetrical CPWR. Thicknesses of the first layer of magnesium fluoride and Au metal film are 500 nm and 40 nm, respectively. Wavelength of the incident light is 632.8 nm, n(BK_7_) = 1.515, n(MgF_2_) = 1.38, n(Au) = 0.3123 + 3.146i; (**c**) Fluorescence intensities against to different concentrations responding to sensor films with different thicknesses of second MgF_2_ layer ranging from 550 nm to 700 nm.

Electric field intensities and propagation length are very important factors for the dark field fluorescence excitation (in which manner the excitation source will not be collected by the detector). For a symmetrical optical waveguide format, electric field distributions depend largely on the thickness of magnesium fluoride in contact with the sensed medium (waveguide layer). To find out the most optimized combinations among the three successive layers of MgF_2_-Au-MgF_2_, different thicknesses for the second layer of MgF_2_ (waveguide layer) are theoretically tested. To acquire a satisfactory resonance feature, we set the thicknesses for the first two layers as 500 nm and 40 nm separately to see how the thickness of the second magnesium fluoride layer will influence the resonance curves and electric field intensity distributions. From the theoretical results given in Figure 4a, it can be figured out that with the thickness increase of the waveguide layer, the resonance angle shifts to a bigger position and the FWHM becomes broader, meanwhile, the CPWR generated evanescent field decays faster (Figure 4b). What is more, CPWR shows longer electric field propagation length (about 2,000 nm) than conventional SPR (about 200 nm) which suggests this structure might be suitable for bead-based fluorescence excitation [25].

In the present experiments, we have prepared four samples with different thicknesses of the second layer of MgF_2_ ranging from 550 nm to 700 nm to test the surface enhanced fluorescence ability of this symmetrical CPWR. Thicknesses of the second layer of MgF_2_ can be varied by changing the amount of MgF_2_ powder used during the vacuum evaporation process.

Cy5 solutions with concentrations of 2, 4, 6, 8, 10 nM are used to acquire fluorescence intensities against concentrations for each set of films. Solutions of different concentrations are pumped into the flow channel with a flow rate of 0.5 mL/min for one minute and then the fluorescence spectra are captured by spectroscopy. We averaged the whole channel to get fluorescence spectra for each concentration and fluorescence intensities are acquired by integrating fluorescence spectra at a wavelength ranging from 650 nm to 677 nm. As expected, combinations of 500 nm-40 nm-600 nm layer thicknesses for MgF_2_-Au-MgF_2_ waveguide structure exhibits biggest fluorescence intensities for each Cy5 concentrations (shown in Figure 4c) and are chosen for further experiments.

### 3.3. Limit of Detection

**Figure 5.**
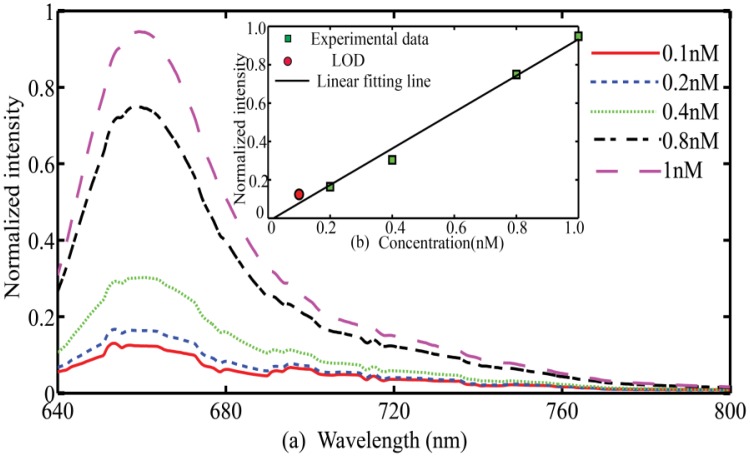
(**a**) Fluorescence spectra for each concentration; (**b**) Plot of normalized fluorescence intensities against solution concentrations.

To test the detection limit (the minimum concentration of the fluorescence solution that we can acquire fluorescence signals) of the system, Cy5 solutions with different concentrations are tested with our system. Figure 5a shows the raw emission spectra corresponding to different concentrations of Cy5 solutions. Layer thicknesses of 500 nm-40 nm-600 nm are used in this series of experiments. From Figure 5b, it can be clearly determined that the concentrations of the Cy5 solutions exhibit an excellent linear relationship with the normalized fluorescence intensities that are integrated at a wavelength ranging from 650 nm to 677 nm. We also find out that the concentration limit of Cy5 solutions that can be detected in this system is 0.1 nM.

### 3.4. Multi-Channel Hyperspectral Fluorescence Analysis

**Figure 6.**
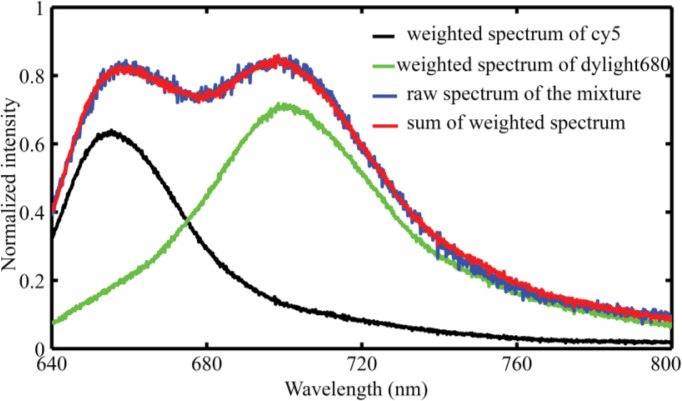
Multivariate analysis of mixed solution containing two spectrally overlapping fluorophores.

Two closely overlapping dyes (Cy5 and Dylight680) are mixed together to test the increased throughput possible with the hyperspectral fluorescence detection method. Firstly, we extract the raw emission spectra of the pure Cy5 solution and absolute Dylight680 labeled secondary antibody. Then the mixed solution containing both Cy5 and Dylight680 is presented in the flowing channel. We acquire the responding spectra of the mixture, shown in Figure 6, where the blue curve represents the raw spectra of the mixture. Multivariate data analysis using linear admixtures of these two dyes applied to both the raw spectra of the mixture and the pure dye solutions to determine the ratios of each dye and the weighted Cy5 (black curve) and Dylight680 (green curve) spectra are shown in Figure 6, respectively. The red curve is a sum of the weighted Cy5 and Dylight 680 spectra. As we can see from Figure 6, the sum curve of the weighted Cy5 and Dylight680 spectra closely matches the raw spectra of the mixture, revealing the fact that we develop a successful multivariate algorithm to model the hyperspectral stack. Besides its advantage in distinguishing closely overlapping fluorescence tags, by spectrally resolving all overall spectra, this system can also identify and eliminate unnecessary artifacts, e.g., the fluorescence emissions from the substrate or contamination. Based on these features, fluorescence encoding technique will be greatly improved by using hyperspectral fluorescence analysis, especially, by combining metal nano-structure enhanced fluorescence technique [26].

## 4. Conclusions

In this work, we have applied a symmetrical structure to afford coupled plasmon-waveguide resonance (CPWR) for the effective excitation of fluorescence. Optimal parameters for combinations among each layer are also investigated theoretically and experimentally in this paper. The hyperspectral fluorescence technique is used for the identification of partially overlapping emission fluorescence tags. The performance characteristics of this novel system are evaluated for Cy5 solutions and a limit of detection of 0.1 nM could be achieved. We record the entire spectra of the solution mixed with Cy5 and Dylight680, ratios of different fluorescence can be acquired accurately by the use of multivariate algorithms. These results show the capability for the detection of more than two tags with partially overlapping emission spectra in one experiment. Besides, a line-monitoring schedule makes it possible for multi-channel detection that greatly enhances the throughput capabilities. On account of these features, this new biosensor scheme based on combinations of both symmetrical coupled plasmon-waveguide resonance excitation and the hyperspectral fluorescence analysis technique is believed to allow multiplex analysis for the applications of biochemical interactions and DNA microarray technology.
